# One Year of *JAHA*

**DOI:** 10.1161/JAHA.112.000129

**Published:** 2013-02-22

**Authors:** Joseph A. Vita

**Keywords:** Editorials

## Introduction

With the February 2013 issue, the *Journal of the American Heart Association* (*JAHA*) begins its second year. *JAHA* was created as a forum for high‐quality cardiovascular and cerebrovascular science and to particularly represent the domains of the 16 Scientific Councils of the American Heart Association (AHA).^1^ As an open access journal, *JAHA* was designed to provide readers with immediate subscription‐free access to the entire content of articles from any device connected to the internet. The launch of *JAHA* was particularly timely, because funding agencies in the United States and the United Kingdom have recently established policies requiring that publicly‐funded research be freely accessible.^2–3^ We have no restrictions on article length and provide unlimited use of color images and video, thus allowing authors to present all aspects of their work. It was our particular goal to provide rapid review and posting of manuscripts. In our first year, we have accomplished many of these goals and have begun to establish our identity within the AHA family of journals.

In 2012, we published 99 original articles and 24 reviews/editorials and had a marked increase in the number of articles per issue during the course of the year (Figure). Articles are reviewed rapidly, with a median time to first decision of 19.5 days, and are posted online within 4 weeks of acceptance. Articles are submitted from around the world: Over 40% of our submissions came from outside the United States, including 8% from Japan, 5% from the United Kingdom, 5% from Canada, and 4% from Germany. As authors become more aware of *JAHA*, we expect further increases in the number of articles published per issue and we are striving to decrease the time for review and online publication.

**Figure 1.**
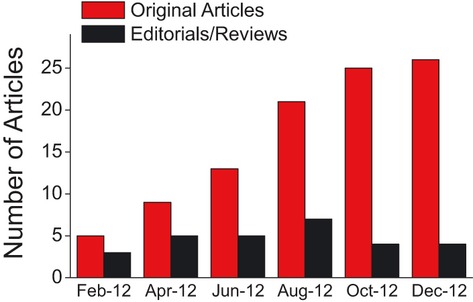
Number of original articles and editorials/reviews published in *JAHA* by month in 2012.

Our published articles covered a broad range of cardiovascular science. About two‐thirds of the original articles reported clinical science and one‐third reported basic science. The most frequent topic categories included coronary heart disease, vascular medicine, heart failure, stroke, and electrophysiology. In line with our goal to support the missions of the AHA and to represent the scientific councils, we also published articles on resuscitation science, pediatric cardiology, health services and outcomes, interventional cardiology, cardiovascular surgery, the kidney in cardiovascular disease, nutrition, and physical activity. We also have been a forum for leadership to comment on the AHA's goals for the cardiovascular health of all Americans.^4^ This broad range of topics was achieved, in part, because our editorial board is composed of representatives from each AHA Council. Board members act as guest editors for papers within their area of expertise and help identify editorialists and authors for review articles.

We have developed close relationships with the other AHA journals. The editors of *Arteriosclerosis*,* Thrombosis*, and *Vascular Biology*,* Circulation Research*,* Hypertension*,* Stroke*, and the *Circulation* family of journals cannot exceed their print and online page budgets, and often must reject highly meritorious papers. *JAHA* has no such limits, because we do not have a print version and because publication costs are borne by authors and/or their institutions and research sponsors. We communicate with the editors of the other AHA journals on a daily basis about such papers. After consideration of the content and reviewer comments, we invite the authors of selected papers to submit their work to *JAHA*. This is appealing to many authors because they are asked to address the comments of the original reviewers and do not have to start over with an entirely new set of editors and reviewers. Since we carefully screen papers before offering the option of submitting to *JAHA*, the final decision can be made quickly and the acceptance rate for invited papers is very high as long as the authors appropriately address the reviewers’ comments.

In our first year, 60% of accepted original articles were referred from another AHA journal. The greatest proportion (64%) was referred from *Circulation*, and the editors of *JAHA* have considered referrals from all ten of the other AHA journals. While it is difficult to generalize, several types of papers were invited. For example, the editors invited a number of basic science papers that made highly novel observations, even though the operative mechanisms were not completely defined. We invited well‐done clinical trials, including adequately‐powered negative studies. We also invited well‐conducted observational and outcomes papers, including a number of Get‐With‐the‐Guidelines studies relevant to the missions of the AHA. Given the research interests of some of the editors, we have been highly interested in translational science, particularly work that examined mechanisms of cardiovascular disease in human subjects. Finally, we invited a number of high‐quality papers that did not achieve priority largely because the topic of research was highly specialized and fell outside the domain of the original AHA journal. The editors emphasize that all invited and direct submissions undergo rigorous peer review. We particularly focus on statistical issues, and all papers undergo review by our Statistical Editor and a team of statistical consultants.

Open access publication is important because it facilitates the dissemination of scientific knowledge. During our first year, we took several steps to take full advantage of the internet and to increase accessibility of our manuscripts beyond simply posting them on the *JAHA* website (http://jaha.ahajournals.org/). All articles are deposited into PubMed Central and we successfully applied to the National Library of Medicine for indexing of *JAHA* on Pub Med. Our publisher developed a user‐friendly iPAD/smartphone app that can be downloaded without cost by searching for “JAHA.” Readers also can learn about and connect to the full text of our articles by following *JAHA* on Twitter (@Jaha_aha) or Facebook (use the direct link http://goo.gl/Fvt9B or search for “JAHA”). In addition to highlighting new articles each week, our Facebook page has interesting additional content, including regular ECG challenges and author profiles.

In summary, we have made considerable progress during our first year. We are publishing an increasing number of outstanding articles that cover a wide range of cardiovascular science. The review process is rapid, and we are working to increase the visibility and accessibility of our papers. Authors working in any domain of cardiovascular research should consider *JAHA* as a journal that can provide rapid review and the widest possible dissemination of their work. Our ultimate goal is to facilitate communication of cardiovascular science that will improve the care of patients with cardiovascular disease and stroke.
